# Retrovision

**DOI:** 10.1038/s44319-024-00087-9

**Published:** 2024-02-08

**Authors:** Howy Jacobs

**Affiliations:** Tampere University, Tampere, Finland

**Keywords:** Careers, History & Philosophy of Science

## Abstract

The Covid pandemic is long past, but its detrimental effects on science still linger.

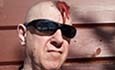

The pandemic is now sufficiently in the rear-view mirror that we can begin to assess its lasting effects on society and the workplace, in particular, how it has impacted science and scientific discourse. Talking with colleagues, it seems that experiences differ widely between institutes and countries; but there are, nevertheless, a few common threads. Some of these issues have recently been highlighted in an excellent op-ed elsewhere (Lewis et al, 2024), so I will dwell here only on a couple of them that are of particular concern to me.

Working from home, at least part-time, has now become widely accepted. Most academics still come to their university campus on some or most days of the week, but many are rarely there at what used to be called ‘unsocial hours’, even if they expect their graduate students to be in attendance from dawn till dusk and beyond—though they frequently aren’t: if the boss isn’t there, there is less pressure to show their own face.

Indeed, with the luxury of no longer having assigned teaching duties or of running my own lab, I have adopted such a pattern of work myself, and find it conducive to productivity, especially whenever I have deadlines to obey. Being single, this is easy for me, while for others it is more of a burden. The huge downside for all of us is that it is far harder to interact casually with colleagues, students, or visitors, other than with those whom we are hosting ourselves.

Those who manage workplaces—and universities are no exception—see this as a welcome, cost-saving development to decrease the amount of rented office space to a bare minimum, with some staff and sometimes even full professors sharing offices or computer terminals. Even then, many university buildings seem to be permanently half-empty. To locate someone, even just to ask a trivial question, can be a fruitless endeavor best replaced with an exchange of email or voicemail messages. Arguably, this can nurture better and more streamlined interactions, assuming people are actually at their workstations somewhere. But, equally, it devalues the human dimension, especially when dealing with someone you’ve never met in person, such as a recently appointed HR manager, janitor, or core-facility technician.

The biggest loss, however, is the coffee-room chit-chat. Even if most of the conversation is about non-scientific matters, these chance encounters are part of what makes us human, and stimulates the creative processes that make for good science. It’s impossible to measure whether those rare flashes of insight that arise from the tangential threads of an unprogrammed discussion compensate for the time wasted on idle gossip. But my overriding feeling is that far more time is wasted on experiments that ‘don’t work’ or at least don’t actually answer the question posed, when students, postdocs, and PIs are not in regular but random collision in the corridor, canteen, and common-room.

A more difficult issue arises regarding seminars and lectures. Almost all universities have reverted to in-person teaching, if with an increased use of online tools and exercises. But research seminars, which are universal in life-science institutes and were once the centrepiece of every week’s activity, still cling to remote or hybrid format in many places. It’s far cheaper than flying someone halfway across the world for a 45-min talk, if they can appear on screen: also without requiring them to spend an extra night or two in a hotel to recover from jetlag or just to recuperate from the exhaustion and stress of incessant travel. Seminar organizers no longer have to bother taking out speakers for dinner, or searching for hosts who are free from family obligations and don’t suffer from a profound aversion to over-priced, fine-dining restaurants.

Nevertheless, I would readily swap ten online seminars for one real one. In fact, when I invite speakers at my own institute, I no longer enable remote participation at all. It detracts from real-life attendance in a way that is disrespectful to an honored guest and embarrassing to the host institution. Flying someone in from abroad to speak to a live audience of 20 persons, even with another 70 online attendees somewhere else—some maybe not really there at all—is simply pointless. And there are many other drawbacks of the hybrid format. Remote participants rarely engage in Q&A, and the resulting lack of interaction somehow deters those actually in attendance from participating in discussion either. Speakers are far more uncomfortable when one or two persons in an audience of 20 doze off than when the somnolent few are diluted by 100 attentive others. Personally, whenever I myself give a talk, I find it far easier to summon and convey my own enthusiasm for the subject in front of a live audience, when I can look directly at real people and gauge from their faces whether I am pitching the material at the right level.

Despite three years of practice and supposedly state-of-the-art equipment and software, there are so many occasions when a hybrid seminar is disrupted because someone in the room can’t be heard or seen by a remote participant or *vice versa*, or the slides don’t appear on cue or some other annoying bug that takes time to fix and detracts from the flow of the talk. Live events can of course suffer disruptions too, such as travel that is messed up by strikes or weather, or double-booked auditoriums, but these are comparatively rare occurrences.

However, reviving the tradition of live seminars can be tricky. Many institutes have experienced a drop-off in attendance that continues, even long after the health precautions about mask wearing and social distancing have disappeared. If PIs are mostly working from home, they are less likely to attend, and if PIs are absent their students and postdocs feel free to do likewise. As a result, the institute comes to feel more like any old workplace, rather than a community of dedicated scholars, committed to learning by the exchange of ideas.

There are several other advantages of hosting a real-life speaker, especially for those, like myself, in relatively remote locations or small research communities. It presents an opportunity for young scientists to meet and interact on a personal level with research experts from elsewhere, who may not be focused on exactly the same scientific problem, but nevertheless have valuable insights to provide. It allows those small or remotely located institutes to showcase their best science, become better known in the outside world, and develop new and potentially fruitful collaborations. Provided the host doesn’t tire out the guest too badly (apologies to those whom I have failed in this regard) it’s also a way for visitors to cross-pollinate their own thinking by talking with scientists at the local venue. One particularly valuable item on such agendas is for the visitor to meet with a group of PhD students over a sandwich lunch, inviting them to present their projects briefly and without slides, as well as discuss career issues and options.

Visiting a new city or country can be a stimulating and enjoyable experience beyond science, much appreciated by seminar speakers, provided the host organization isn’t too stingy about covering the visitor’s (or hosting scientist’s) expenses. It’s again a bit embarrassing to be told that ‘our funder doesn’t cover the cost of alcoholic drinks’, or ‘I would love to have brought along two of my postdocs but the university won’t allow it’. All the advantages are lost if remote or hybrid seminars replace the real thing, even if there seem to be more attendees, at least ‘on paper’.

Finally, there are few mechanisms in place to allow for the lost years in people’s CVs to be taken into account. We have finally started to make due allowance for career breaks, such as professional training, military service and childbirth, in judging applications for jobs, grants or fellowships. But low productivity due to the pandemic is largely ignored, especially because it isn’t universal. Many scientists, young or old, were unable to work effectively for more than 2 years, due to local or personal circumstances, the psychological effects of lockdowns or other restrictions, or just the nature of their projects. Others were able to find ways to continue more or less as normal. It is vital that we don’t further penalize those who suffered the most, by denying them the opportunities they deserve.
